# Assessment of tumour size in PET/CT lung cancer studies: PET- and CT-based methods compared to pathology

**DOI:** 10.1186/2191-219X-2-56

**Published:** 2012-10-03

**Authors:** Patsuree Cheebsumon, Ronald Boellaard, Dirk de Ruysscher, Wouter van Elmpt, Angela van Baardwijk, Maqsood Yaqub, Otto S Hoekstra, Emile FI Comans, Adriaan A Lammertsma, Floris HP van Velden

**Affiliations:** 1 Department of Radiology & Nuclear Medicine, VU University Medical Center, PO Box 7057, Amsterdam, 1007MB, The Netherlands; 2 Department of Radiation Oncology (Maastro Clinic), GROW School for Oncology and Developmental Biology, Maastricht University Medical Center, Maastricht, the Netherlands

**Keywords:** Tumour delineation, Tumour diameter, FDG PET, Non-small cell lung cancer

## Abstract

**Background:**

Positron emission tomography (PET) may be useful for defining the gross tumour volume for radiation treatment planning and for response monitoring of non-small cell lung cancer (NSCLC) patients. The purpose of this study was to compare tumour sizes obtained from CT- and various more commonly available PET-based tumour delineation methods to pathology findings.

**Methods:**

Retrospective non-respiratory gated whole body [^18^F]-fluoro-2-deoxy-D-glucose PET/CT studies from 19 NSCLC patients were used. Several (semi-)automatic PET-based tumour delineation methods and manual CT-based delineation were used to assess the maximum tumour diameter.

**Results:**

50%, adaptive 41% threshold-based and contrast-oriented delineation methods showed good agreement with pathology after removing two outliers (R^2^=0.82). An absolute SUV threshold of 2.5 also showed a good agreement with pathology after the removal of 5 outliers (R^2^: 0.79), but showed a significant overestimation in the maximum diameter (19.8 mm, p<0.05). Adaptive 50%, relative threshold level and gradient-based methods did not show any outliers, provided only small, non-significant differences in maximum tumour diameter (<4.7 mm, p>0.10), and showed fair correlation (R^2^>0.62) with pathology. Although adaptive 70% threshold-based methods showed underestimation compared to pathology (36%), it provided the best precision (SD: 14%) together with good correlation (R^2^=0.81). Good correlation between CT delineation and pathology was observed (R^2^=0.77). However, CT delineation showed a significant overestimation compared with pathology (3.8 mm, p<0.05).

**Conclusions:**

PET-based tumour delineation methods provided tumour sizes in agreement with pathology and may therefore be useful to define the (metabolically most) active part of the tumour for radiotherapy and response monitoring purposes.

## Background

Positron emission tomography (PET) is a functional imaging modality that provides information about metabolism, physiology and molecular biology of tumour tissue. ^18^F]-fluoro-2-deoxy-D-glucose (FDG) is the most widely used radiotracer that provides information on glucose metabolism. There is an increasing interest in using FDG PET not only to determine FDG uptake but also to determine the location and extent of the metabolic active part of the tumour. PET is being explored as a tool for e.g. the definition of the gross tumour volume (GTV), location of the metabolic active part of the tumour for radiation oncology or monitoring response during chemotherapy [1-3]. For radiation treatment planning, the GTV is defined mainly on computed tomography (CT), which provides anatomical image data. CT imaging, however, has low contrast for soft tissue, making it difficult to differentiate between tumour and normal tissue due to their similar electron density values. It is hypothesised that PET could improve accuracy of GTV definition for radiation treatment planning [4]. Accurate GTV definition is vital in order to generate a highly conformal radiation dose distribution, thereby sparing surrounding normal tissue and allowing a higher radiation dose to the most active part of the tumour. In addition to improve the accuracy of GTV definition, PET can indicate areas within the tumour that are metabolically more active or malignant, which may be used to define areas that need an additional radiotherapy boost [5].

Another important application of FDG PET is its use for the assessment of (early) treatment response and/or as a prognostic factor. So far, these applications have been mainly explored by studying FDG uptake quantitatively by means of standardised uptake values (SUV). Nevertheless, other parameters, such as the metabolic volume or total lesion glycolysis (product of SUV and metabolic volume), may provide additional valuable information both as prognostic value as well as for treatment response monitoring. Recently, metabolic tumour volume or maximum metabolic tumour diameter have been shown to be independent prognostic factors for oesophageal cancer but only when accurate PET-based tumour delineation methods are used [6]. Moreover, for response monitoring studies it is important to know whether a difference in SUV or metabolic tumour volume in successive scans represents a true response or represents methodology-related variability. Therefore, accurate and reproducible metabolic volume delineation is important.

Several (semi-)automatic PET-based tumour delineation methods have been studied previously [7-12]. It is generally accepted that pathology is the gold standard and should be used for validation of tumour delineation methods. However, only a limited number of (lung) tumour studies [4,13-17] have been performed that compare data obtained from (semi-)automatic PET-based tumour delineation methods with pathological data. Therefore, the primary purpose of this study was to compare measured tumour sizes obtained from a broad spectrum of (more commonly available) PET-based tumour delineation methods to pathology findings. In addition, CT-based tumour size assessments were compared with pathology.

## Methods

### Patients, PET/CT and pathology

Included in this study were 19 consecutive patients (8 females and 11 males; weight 75±14 kg, range 42–100 kg) with histological proven non-small cell lung cancer, who had undergone a diagnostic whole body PET/CT scan and underwent a surgical resection of their primary lung tumour in the period from December 2003 to December 2004. All patients gave written informed consent prior to inclusion and the study was approved by the Medical Ethics Review Board of the Maastricht University Medical Center.

FDG data were acquired using a whole-body PET/CT scanner (Biograph, Somatom Sensation 16; Siemens, Erlangen, Germany). FDG was administered as an intravenous bolus (365±62 MBq). For each patient, plasma glucose levels were measured (mean 5.7±2.1 mmol·L^-1^, range 4.1-12.0 mmol·L^-1^) and all patients fasted for at least 6 h before scanning. PET data were reconstructed using ordered subsets expectation maximisation with 4 iterations and 18 subsets, followed by 5 mm full width at half maximum (FWHM) Gaussian smoothing, resulting in an image resolution of about 6.5 mm FWHM. All PET images had a matrix size of 128×128×178, corresponding to a pixel size of 5.31×5.31×5.00 mm^3^. CT data had an image matrix size of 512×512×178, corresponding to a pixel size of 0.98×0.98×5.00 mm^3^. CT data were used to correct for tissue attenuation. More acquisition and reconstruction details can be found elsewhere [4].

All patients underwent surgical resection of their primary tumour after approximately 47 d (range: 7 to 112 d). Directly after surgical resection, the maximum diameter of this tumour was measured by macroscopic examination in three dimensions using a calliper. Shrinkage of the tumour, estimated to be around 10%, was not considered as no preservation, fixation or inflation was applied prior to the diameter measurements. The obtained maximal diameters ranged from 1.5 to 7.0 cm (mean: 4.0±1.8 cm). The primary tumours were located in the superior (n=8), middle (n=1) or inferior (n=10) lobe.

### Data analysis

Maximum tumour diameters were measured for each primary lung tumour volume. These tumour volumes were obtained using six different types of PET-based (semi-)automatic tumour delineation volume of interest (VOI) methods:

1 Fixed threshold of 50% and 70% of maximum voxel value (VOI^50^, VOI^70^) [18].

2 Adaptive threshold range of 41-70% of maximum voxel value (VOI^A41^, VOI^A50^, VOI^A70^). Same as above, except that it adapts the threshold relative to the local average background [18].

3 Contrast-oriented method (VOI^Schaefer^) [9]. This method was recalibrated for the image characteristics used.

4 Background-subtracted relative-threshold level (RTL) method (VOI^RTL^). This method is an iterative method based on a convolution of the point-spread function that takes into account the differences between various sphere sizes and the scanner resolution [7].

5 Gradient-based watershed segmentation method. This method uses two steps before calculating the VOI. First, this method calculates a gradient image on which a ‘seed’ is placed in the tumour and another one in the background. Next, a watershed (WT) algorithm is used to grow the seeds in the gradient basins, thereby creating boundaries on the gradient edges. In our study, two different types of gradient basins were used. The first approach, indicated by Grad^WT1^, assigns all voxels on the edge between the tumour and the background to the tumour [10-12]. The second approach uses an upsampled image to ensure less effects of sampling. In addition, voxels that indicate an edge between the tumour and the background are given to either the tumour or the background depending on which region has a value closest to the edge voxel value [12].

6 Absolute SUV (SUV^2.5^). Normalised (SUV) voxel intensities at a chosen absolute threshold (2.5) are used to delineate tumour [19].

More details on the methods used can be found in [10-12]. In addition to PET-based tumour delineation, the tumour was also delineated manually on the CT image by an expert physician. Window and level settings were varied according to the expertise by the expert physician. The volume delineated on CT covered the whole primary tumour.

### Statistical analyses

For the first analysis, the measured maximum tumour diameters were compared with the maximum diameter obtained from pathology. The maximum diameter was obtained from the derived tumour volume by measuring diameters in all possible directions, possibly including spans of regions inside the tumour that are e.g. necrotic or cystic. For each tumour delineation method, mean, median, minimum and maximum values of maximum diameter of the primary tumours were reported. In addition, for each delineation method, correlation of maximum diameter with corresponding pathology data was determined.

As suggested by Wanet et al. [14], we also performed a second analysis that uses a logarithmic transformation of the data to reduce the magnitude of both skewness and kurtosis of the volume distributions, so a nearly Gaussian distribution of the data was obtained. For this analysis, the difference in logarithmic transformed data was calculated as $\ln\left(diameter_{\mathrm{VOI}}\right)-\ln\left(diameter_{pathology}\right)$, where diameter_VOI_ is the maximum tumour diameter of either manual CT delineation or various PET-based tumour volume delineation methods and diameterpathology is the maximal pathological diameter. Correspondence in maximal diameter for each tumour delineation method was also evaluated by Bland-Altman plot analysis. P-values were calculated using the two-tailed Wilcoxon signed-rank test between maximal diameters obtained from PET- or CT-based delineation and pathology. P-values, which were lower than 0.05, were considered statistically significant. Outliers were identified visually as VOIs that showed an unrealistically large measured tumour volume.

## Results

During analyses VOI^50^, VOI^A41^, VOI^Schaefer^ and Grad^WT1^ showed two outliers for which the generated VOI resulted in unrealistically large volumes, as assessed visually. In addition, SUV^2.5^ showed five outliers that had an unrealistically large measured volume. Therefore, all means and correlation analyses were corrected for these outliers.

**Figure 1:**
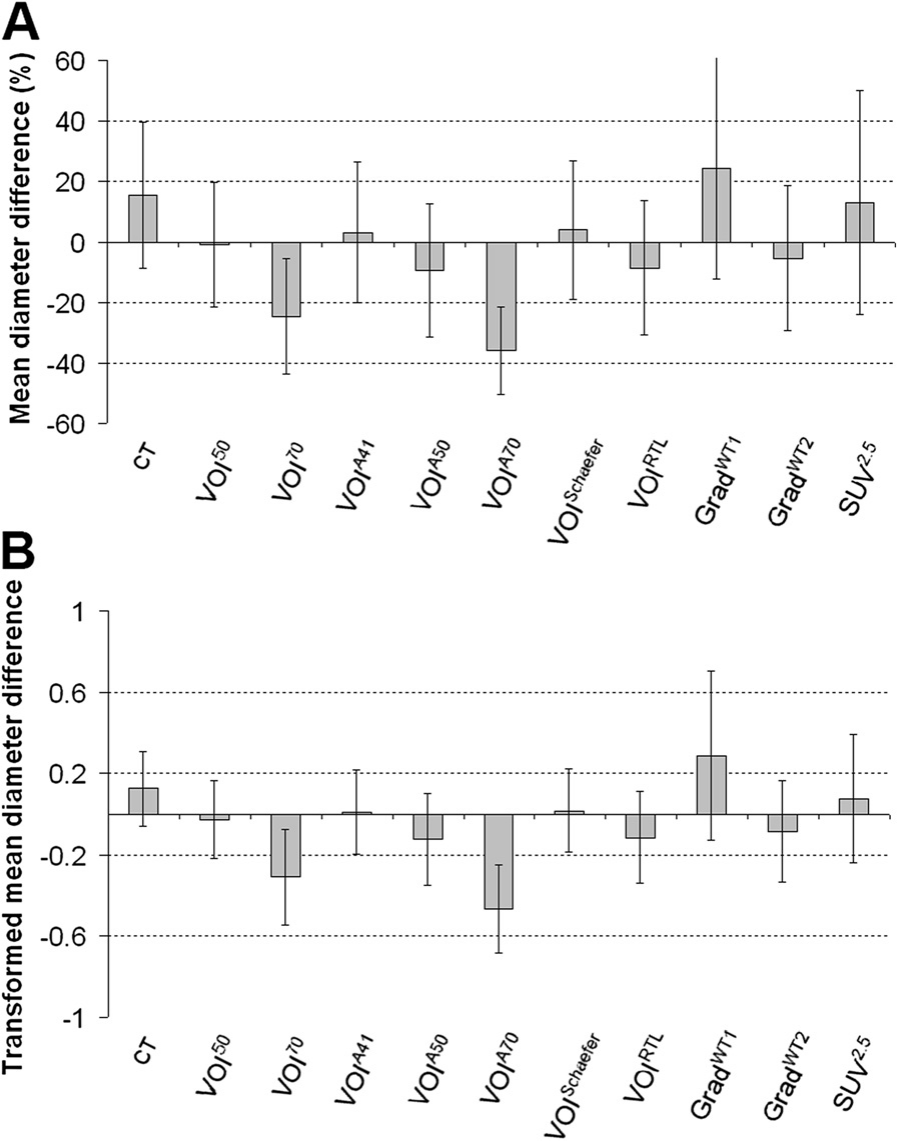
**Mean differences between CTand PET derived maximumtumour diameters and correspondingpathology data.** Percentage mean difference (**A**) or logarithmically transformed mean difference (**B**) between maximum tumour diameters derived from both manually delineated tumours on CT and several (semi-)automatic PET-based delineation methods and corresponding data from pathology. Error bars represent standard deviation.

Figure 1 shows mean differences between CT and PET derived maximum tumour diameters and corresponding data obtained from pathology. Except for VOI^70^, VOI^A70^ and Grad^WT1^ (−24.6, -35.9 and 24.4%, respectively), mean differences were smaller for all PET-based methods (−9.4 to 13%) than for manual CT delineation (15%). VOI^50^, VOI^A41^ and VOI^Schaefer^ provided estimates of the maximum diameter (−0.8, 3.0 and 3.9%, respectively) that were closest to those obtained from pathology.

An identical trend can be seen in Table 1. The median diameter (across tumours) obtained from VOI^50^ was similar to that obtained from pathology (i.e. 0.6 mm difference; p=0.984). For CT-based delineation, corresponding differences in median diameter were significantly larger (3.8 mm difference; p=0.002). In addition, a significant overestimation of median diameter compared to pathology was found for SUV^2.5^ and Grad^WT1^ (19.8 and 13.8 mm difference, respectively; p<0.05). Some other PET-based methods (i.e. VOI^A41^, VOI^A50^, VOI^Schaefer^, VOI^RTL^ and Grad^WT2^) provided results that were not statistically different from pathology (p>0.10). However, median diameters obtained from VOI^A50^, VOI^RTL^ and Grad^WT2^ were slightly smaller (<4.7 mm differences) than those obtained from pathology. In contrast, only small differences in median diameter obtained from VOI^A41^ and VOI^Schaefer^ (2.0 and 3.3 mm difference, respectively) were observed when compared with pathology.

**Table 1:** **Mean, median, minimum and maximum values of maximum tumour diameter as obtained with different methods**

**Method**	**Maximum diameter (mm)**				
	**Mean**	**Median**	**Min**	**Max**	**p-value**^**a**^
Pathology	40.0	35.0	15.0	70.0	-
CT	48.1	38.8	17.3	137.0	0.002
PET					
VOI^50^	39.4^b^	35.6	15.5	333.6	0.984
VOI^70^	30.9	24.1	11.3	71.4	<0.001
VOI^A41^	41.3^b^	37.0	15.5	330.6	0.623
VOI^A50^	37.2	30.3	12.5	92.0	0.104
VOI^A70^	26.6	22.5	10.6	62.3	<0.001
VOI^Schaefer^	41.7^b^	38.3	15.5	333.6	0.568
VOI^RTL^	37.9	33.8	10.6	92.8	0.113
Grad^WT1^	50.0^b^	48.8	32.5	83.2	0.003
Grad^WT2^	36.2	32.3	19.5	79.0	0.156
SUV^2.5^	44.3^b^	54.8	12.5	643.4	0.009

^a^Significance of the difference between tumour delineation method and pathology.

^b^Without outliers (2 for VOI^50^, VOI^A41^, VOI^Schaefer^ and Grad^WT1^, 5 for SUV^2.5^).

Table 2 shows the correlation of maximum diameters obtained with various PET- and CT-based tumour delineation methods with pathology data. All PET-based methods provided reasonable correlations (R^2^>0.62; slope 0.69-1.16), except for Grad^WT1^ (R^2^=0.43; slope 1.12). Although VOI^A70^ showed good correlation (R^2^=0.81), comparable with VOI^50^, VOI^A41^ and VOI^Schaefer^, it resulted in an underestimation of the maximum diameter (slope 0.69).

**Table 2:** **Linear regression data of maximum diameter obtained using several delineation methods and pathology**

**Delineation method**	**R**^**2 a**^	**Slope**^**a**^
CT	0.77	1.25
PET		
VOI^50 b^	0.82	1.00
VOI^70^	0.73	0.79
VOI^A41 b^	0.82	1.05
VOI^A50^	0.75	0.95
VOI^A70^	0.81	0.69
VOI^Schaefer b^	0.83	1.06
VOI^RTL^	0.77	0.97
Grad^WT1 b^	0.43	1.12
Grad^WT2^	0.62	0.88
SUV^2.5 b^	0.79	1.16

^**a**^With intercept set to 0.

^b^Without outliers (2 for VOI^50^, VOI^A41^, VOI^Schaefer^, and Grad^WT1^, 5 for SUV^2.5^).

**Figure 2:**
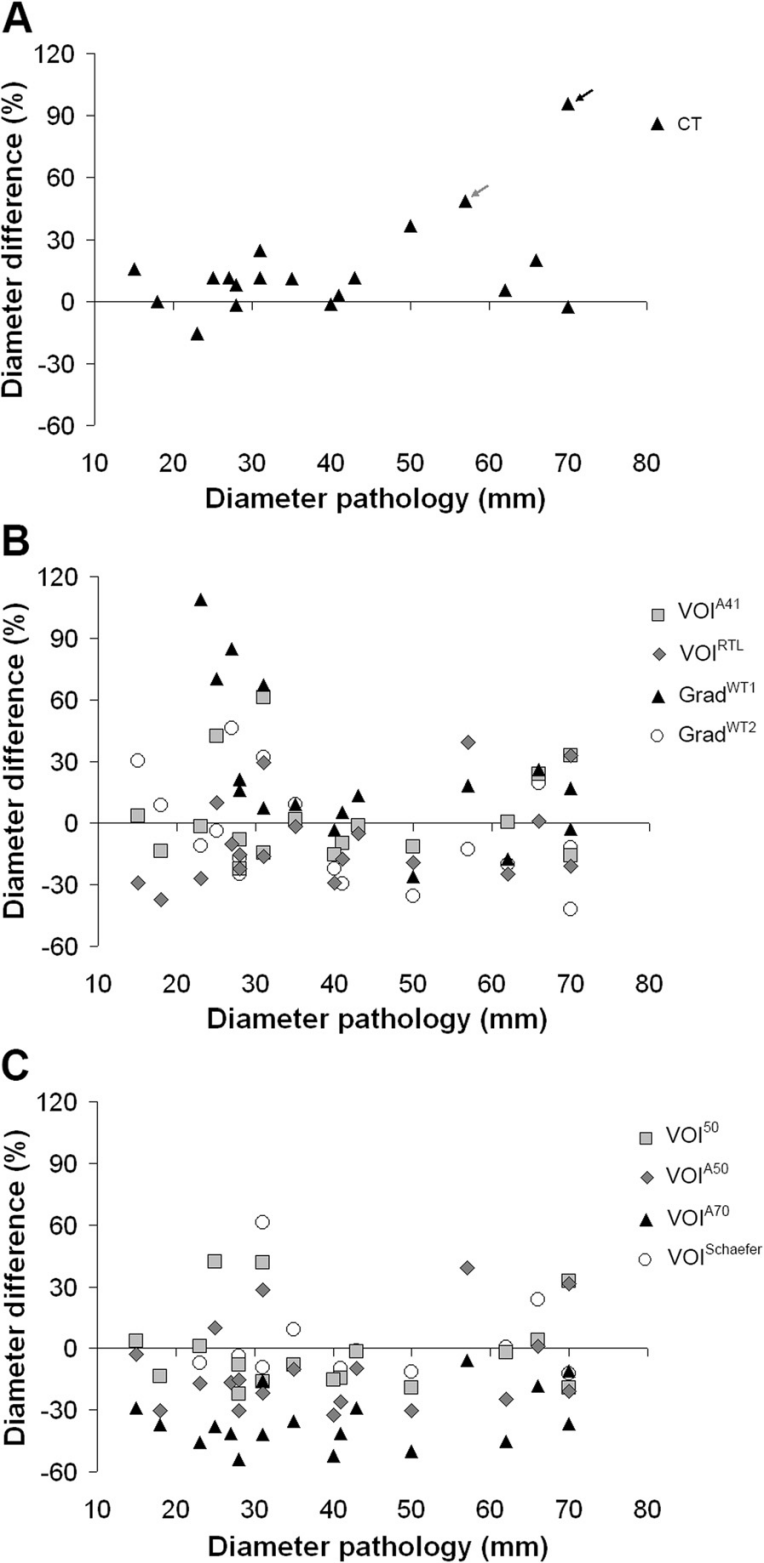
**Differences in maximum diameterderived from tumour delineationmethods and that obtainedfrom pathology.** Difference in maximum tumour diameter between manual CT delineation (**A**) or various PET-based tumour volume delineation methods (**B-C**) and pathological size as function of pathological diameter. The arrows indicate two patients with heterogeneous lung tumours. Note that two outliers each from VOI^50^, VOI^A41^, VOI^Schaefer^ and Grad^WT1^ fall outside the range of the figure (i.e. >220%).

**Figure 3:**
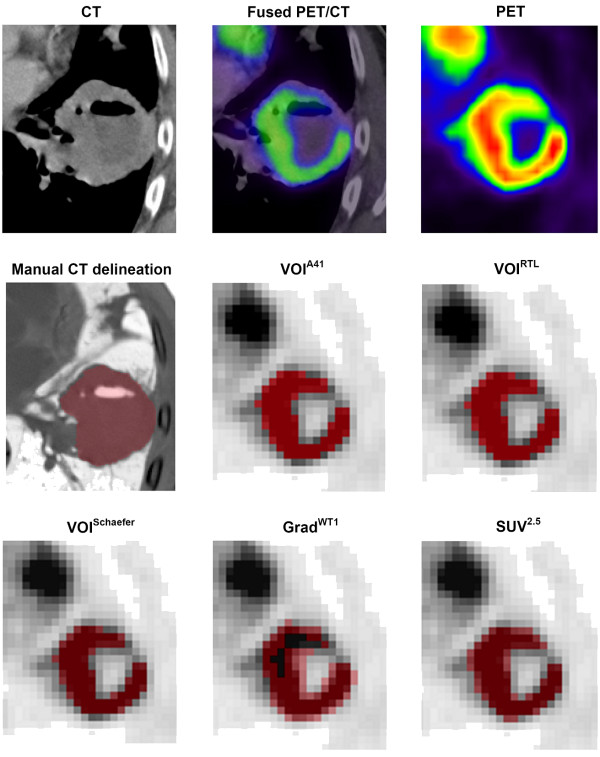
**Example volumes of interest(VOIs) obtained with variousdelineation methods for thelarge primary tumour.** Example of axial CT, fused PET/CT and PET images as well as VOIs obtained from CT-based and various PET-based delineation methods (VOI^A41^, VOI^RTL^, VOI^Schaefer^, Grad^WT1^ and SUV^2.5^) for a lung tumour with heterogeneous FDG uptake. Note that the panes of fused PET/CT and PET images are interpolated to match the CT. All other panes use the original image and voxel sizes.

**Figure 4:**
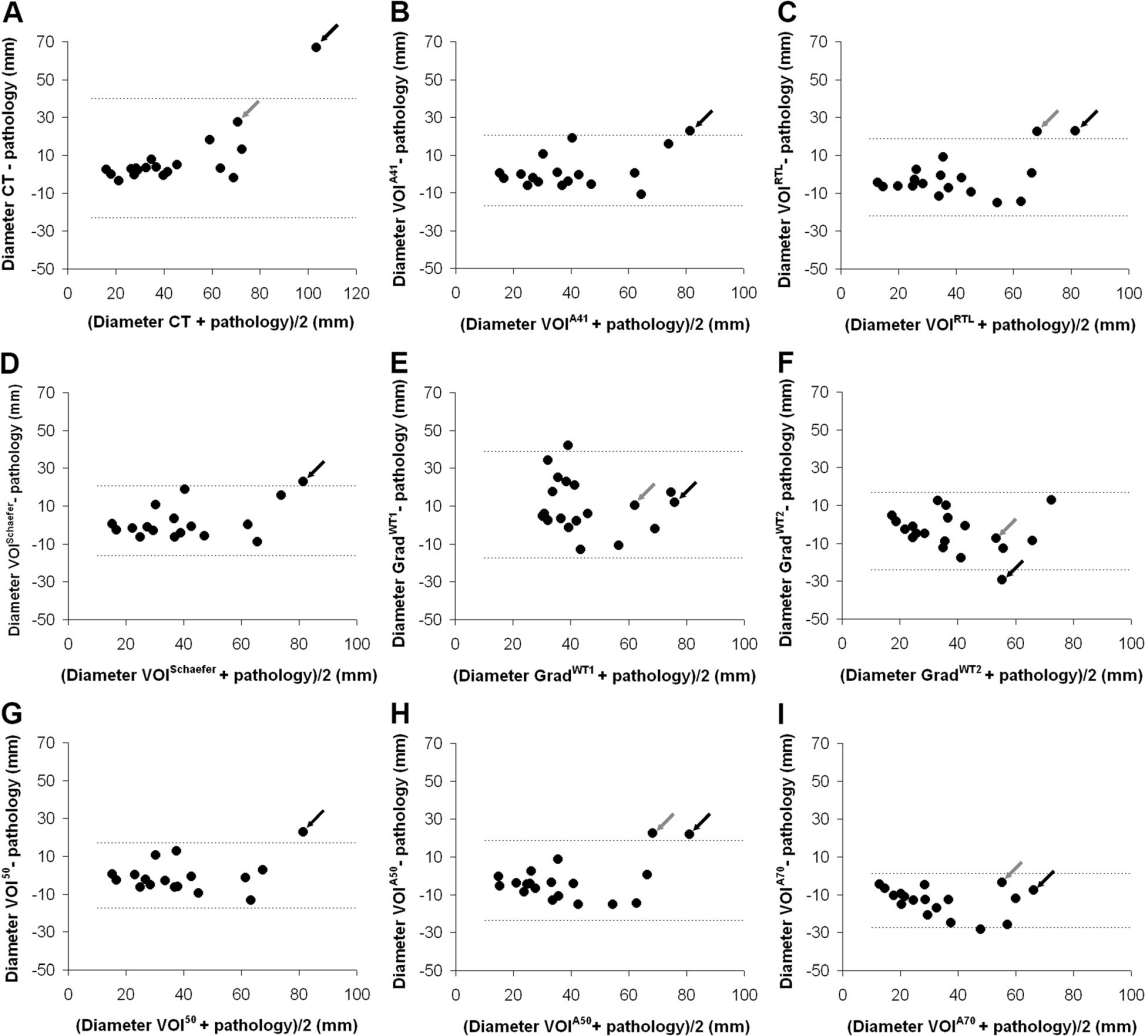
**Bland-Altman analysis.** Bland-Altman plots of the maximum tumour diameter obtained using CT- (**A**) and various PET-based (**B-I**) delineation methods versus those derived from pathology. Dashed lines represent limits of agreement (±1.96×SD of the mean). Arrows indicate two patients with large heterogeneous lung tumours. Note that two outliers from VOI^50^, VOI^A41^ and VOI^Schaefer^ fall outside the range of the figure (i.e. >110 mm).

Figure 2 shows differences in maximum diameter derived from various PET-based tumour delineation methods and that obtained from pathological specimens. Also included is the difference for manual CT delineation. For all patients, most PET-based tumour delineation methods could measure the maximum tumour diameter with moderate accuracy. However, a bad correspondence between diameters obtained with pathology and Grad^WT1^ was found for small tumours (≤2.5 cm diameter). When excluding these small tumours, mean difference (15.7%) and correlation (R^2^=0.60, slope 1.09) between diameters obtained with Grad^WT1^ and pathology moderately improved. Manual CT delineation showed a large maximum diameter for two tumours with heterogeneous FDG uptake (indicated by two arrows: Figure 2A). Figure 3 shows VOIs obtained with various delineation methods for the large primary tumour that is indicated in other figures by a black arrow. For the tumour indicated by the black arrow, only Grad^WT2^ also showed a large difference in diameter (−41.8%: Figure 2B). However, for the tumour indicated by the grey arrow, three methods (VOI^50^, VOI^A41^ and VOI^Schaefer^) showed a large difference in diameter (>485% difference). The same trend was seen in Bland-Altman plots, as shown in Figure 4. For all methods, most data points were within the limits of agreement, i.e. ±1.96×SD of the average), except for patients with large heterogeneous tumours (indicated by the arrows) and tumours located near high uptake regions (i.e. mediastinum or heart). For Grad^WT1^, however, one data point from a patient with a small primary tumour (1.8 cm diameter) was not within the limits of agreement.

## Discussion

Obtaining accurate (metabolic) tumour boundaries may be important for treatment planning in radiotherapy and/or for use as prognostic factor and/or to monitor response during therapy, and may therefore have a direct impact on clinical outcome.

Tumour delineation methods are only suited for radiotherapy planning purposes if they correspond well with pathology. The present results indicate that VOI^50^, VOI^A41^ and VOI^Schaefer^ show good agreement with pathology after removing two outliers (R^2^: 0.82, slope: 1.00-1.06, Figure 1). These outliers were located closely to high uptake regions, e.g. mediastinum and heart. Only those tumour delineation methods should be selected for radiotherapy planning purposes if they are able to distinguish between these adjacent normal tissues and the tumour. VOI^A50^, VOI^RTL^ and Grad^WT2^ did not show any outliers, provided only small, non-significant differences in maximum tumour diameter (<4.7 mm, p>0.10), and showed fair correlation (R^2^>0.62, slope 0.88-0.97) with pathology (Tables 1 and 2). These results correspond with those of previous studies [4,13], in which small differences in maximum tumour diameter between an adaptive threshold method and pathology were reported [13], as well as good correlation between maximum diameters obtained from a percentage threshold-based method and pathology [4]. The latter study also showed a reduction in inter-observer variability when PET-based (semi-)automatic tumour delineation methods were used. Grad^WT1^ showed only moderate agreement with pathology (R^2^: 0.43) and overestimated the maximum diameter. In addition, a poor agreement obtained with pathology and Grad^WT1^ was found for small tumours (≤2.5 cm diameter). As the sizes of the small tumours are less than three times the full-width-at-half-maximum, the influence of partial volume effects may be relatively high, causing this poor agreement with pathology. By applying modifications to the algorithm (i.e. Grad^WT2^), the method showed more accurate tumour sizes (R^2^: 0.62). These findings are in line with a previous study that compared volumes derived with a gradient-based method to pathology [14]. SUV^2.5^ showed a good agreement with pathology after the removal of 5 outliers (R^2^: 0.79), but showed a significant overestimation in the maximum diameter (19.8 mm, p<0.001). Due to the large number of outliers and large overestimation in diameter size, it is not recommended to use this method for radiotherapy planning purposes.

Assessment of change in tumour size is important when monitoring tumour response during therapy. Although VOI^A70^ showed a large systematic underestimation (−36%) of diameter, this method was the most precise (smallest SD and coefficient of variation (COV, $\frac{SD}{Mean}\times 100\%$) of 14 and 40%, respectively (Figure 1). For all other methods, SD and COV ranged from 19 to 188% and from 77 to 448%, respectively). In addition, good correlation (R^2^=0.81) between maximum diameters obtained from this method and pathology was observed (Table 2). Therefore, VOI^A70^ may be a good method for response monitoring in which relative changes in (more active part of) metabolic volume are considered.

Wu et al. [16] showed that CT-based delineation provided better correlation (R^2^=0.87) with pathology than PET-based percentage threshold methods that did not correct for background activity (R^2^=0.77). This is in contrast to the present study, where CT-based delineation provided a slightly lower correlation of maximum diameter with pathology than VOI^50^ (R^2^: 0.77 and 0.82, respectively). In addition, CT-based delineation showed a moderate overestimation of maximum diameter compared with pathology (slope: 1.25 and 1.00 for CT and VOI^50^, respectively). Despite the good correlation, a drawback of manual CT delineation is the requirement of both a high resolution image (i.e. not a low dose CT) and an experienced observer. Even if delineation is performed by an experienced observer, manual CT delineation suffers from substantial interobserver variation [20]. In addition, accuracy of manual CT delineation was shown to be dependent on the colour window settings used [15,16]. Moreover, several conditions including chronic obstructive lung disease (COPD), cavitation, pleural fluid, necrosis, atelectasis and mucus plugs, which all occur frequently in lung cancer patients, obscure the exact boundaries of the tumour on CT inducing errors in measured tumour volume and/or diameter size. In the present study, CT delineation has been performed by only one expert physician. Although this may weaken the strength of any correlation with pathology, the results of this study were consistent with the results from another study where manual CT delineation was performed by two experienced observers [17].

For the patient shown in Figure 3, large differences in diameter were observed between CT- and PET-based methods. In this case, the primary tumour was located close to another suspicious mass within the lymph node. In addition, the primary tumour showed heterogeneous FDG uptake, and both air-containing cavitation and fluid level on the CT scan. Note that, for this typical example, the measured tumour volume obtained using PET-based delineation methods excluded this (non-metabolic) necrotic and cystic centre that was included in manual CT delineation. However, also note that this (non-metabolic) necrotic and cystic centre was included in all maximum diameter calculations. For this tumour, the maximum diameter obtained from PET-based methods was closer to that of pathology than for corresponding CT delineation, further illustrating the conceptual differences between *anatomical* (CT) and *metabolic* (PET) volumes. As previously suggested [13], CT-based delineation is unable to differentiate between high and low activity regions, and the use of PET can assist in quantifying and visualising heterogeneous tracer uptake across the tumour and PET-based delineation may be useful to define the most active part of the tumour.

Some factors might limit accurate delineation of tumour volumes and corresponding maximum diameters using the commonly available PET-based (semi-)automatic tumour delineation described in this article. First, primary lesions could be surrounded by high uptake regions, e.g. from suspected locoregional metastases, heart and spine. Therefore, application of tumour delineation methods might be more valid for peripheral tumours and less valid for more centrally located tumours, unless a (manually adjustable) bounding box around the tumour is used to prevent delineation of surrounding high uptake regions. Second, the metabolic volume of tumours could show heterogeneous tracer uptake that has been shown to have an impact on threshold-based delineation methods [17]. Moreover, tumours located in the thorax could be affected by respiratory motion. However, a good correlation between pathology and PET data is observed in the present study, which might indicate that lung tumors might not be strongly affected by these effects (at least not in this study). Nevertheless, a slight mismatch between PET and CT data can be observed in Figure 3. Fourth, it should be noted that trends observed in the present study may only be valid for primary lung tumours. For other locations, the local background surrounding the tumour is different, which could have an effect on the performance of the tumour volume delineation methods evaluated [11]. Finally, tumour delineation methods are affected by several factors, such as scanner type, radiotracer, image noise and tumour characteristics [10,11]. So, additional evaluations with pathology, and/or optimisation of systems or tumour delineation methods may be required for other PET/CT systems.

The present study showed some potential methodological limitations that might have influenced the results. First, it should be noted that deformations could occur between *in-vivo* CT imaging and *ex-vivo* pathology due to the softness of lung tissue [21]. The method used in the present study involved no inflation of the tissue after resection nor other deformation compensation techniques. All tumours were measured directly after surgery, without using preservation. Inflation is required to find the exact position of the lung tumour inside of the lung. However, inflation is expected to influence mostly the surrounding lung tissue, as the tumours imaged in this study showed a relatively solid mass. The purpose of the current study was not to determine the exact position, but to measure the maximum diameter of the tumour. In addition, the results of the present study are in line with Siedschlag et al. [21] where inflation was used. Therefore, deformations of the tumour after resection are presumed to be negligible. However, ideally, a CT scan of the excised tumour should have been made to confirm that no deformations occurred. Second, no pathological data on the volume of the primary tumour was available. Therefore, only a comparison with maximum tumour diameter was made rather than with volume. Finally, it should be noted that in this study pathological correlation is available only for resectable lung tumours. However, the majority of patients that will receive radiotherapy suffer from unresectable lung tumours for which accurate tumour volume delineation is critical for treatment. However, obtaining the true volumes for this kind of tumour will remain a challenge yet to be solved.

## Conclusions

The maximum diameter derived from CT-based delineation was overestimated compared to pathology, especially at large tumour diameters. PET-based tumour delineation methods provided maximum diameter sizes in closer agreement with pathology. The PET-based 50%, adaptive 41% threshold-based and contrast-oriented (Schaefer) methods seem to be best suited for assessing tumour sizes (of the metabolically most active part) of primary lung tumours, as it provides the best correspondence with pathology data. However, these methods could show a potential difficulty when located close to high uptake regions. Despite only a non-significant small underestimation compared to pathology data, PET-based adaptive 50%, relative threshold level and gradient-based methods could distinguish between the tumour and these adjacent high uptake normal tissues, and are therefore recommended for radiotherapy purposes. An adaptive 70% threshold-based method may be best suited for response monitoring, as it provides the best precision and best correlation with pathology derived size without suffering from outliers.

## Competing interests

The authors declare that they have no competing interests.

## Authors’ contributions

PC performed data analysis and data interpretation, and is the main author of the manuscript. RB performed a study design, implemented some of the tumor delineation methods, contributed to the intellectual content (supervision) and reviewed and approved the final content of the manuscript. DdR provided PET image data and critically reviewed the manuscript. WvE provided the data and reviewed the manuscript. AvB collected the data and reviewed the manuscript. MY implemented some of the tools to perform tumor delineations and critically reviewed manuscript. OSH reviewed the manuscript and approved its final content. EFIC performed manually CT delineation and reviewed the manuscript. AAL reviewed the manuscript, contributed to the intellectual content (supervision) and approved the final content of the manuscript. FHPvV performed data interpretation, implemented some of the tumor delineation methods, assisted in drafting the manuscript and supervised the project. All authors reviewed the collected data and interpretation, provided feedback for further research during the study and approved the final submitted version of this manuscript.

## References

[B1] Erdi YE The use of PET for radiotherapy Current Medical Imaging Reviews 2007 3 3 16

[B2] Fletcher JW Djulbegovic B Soares HP Siegel BA Lowe VJ Lyman GH Coleman RE Wahl R Paschold JC Avril N Recommendations on the use of 18F-FDG PET in oncology J Nucl Med 2008 49 480 508 10.2967/jnumed.107.047787 18287273

[B3] Wahl RL Jacene H Kasamon Y Lodge MA From RECIST to PERCIST: Evolving Considerations for PET response criteria in solid tumors J Nucl Med 2009 50 Suppl 1 122S 150S 19403881 10.2967/jnumed.108.057307 PMC2755245

[B4] van Baardwijk A Bosmans G Boersma L Buijsen J Wanders S Hochstenbag M van Suylen RJ Dekker A hing-Oberije C Houben R PET-CT-based auto-contouring in non-small-cell lung cancer correlates with pathology and reduces interobserver variability in the delineation of the primary tumor and involved nodal volumes Int J Radiat Oncol Biol Phys 2007 68 771 778 10.1016/j.ijrobp.2006.12.067 17398018

[B5] Petit SF van Elmpt WJ Oberije CJ Vegt E Dingemans AM Lambin P Dekker AL De RD [(18)F]fluorodeoxyglucose Uptake Patterns in Lung Before Radiotherapy Identify Areas More Susceptible to Radiation-Induced Lung Toxicity in Non-Small-Cell Lung Cancer Patients Int J Radiat Oncol Biol Phys 2010 10.1016/j.ijrobp.2010.06.016 20884128

[B6] Hatt M Visvikis D Albarghach NM Tixier F Pradier O Cheze-Le RC Prognostic value of 18F-FDG PET image-based parameters in oesophageal cancer and impact of tumour delineation methodology Eur J Nucl Med Mol Imaging 2011 38 1191 1202 10.1007/s00259-011-1755-7 21365252

[B7] van Dalen JA Hoffmann AL Dicken V Vogel WV Wiering B Ruers TJ Karssemeijer N Oyen WJ A novel iterative method for lesion delineation and volumetric quantification with FDG PET Nucl Med Commun 2007 28 485 493 10.1097/MNM.0b013e328155d154 17460540

[B8] Geets X Lee JA Bol A Lonneux M Gregoire V A gradient-based method for segmenting FDG-PET images: methodology and validation Eur J Nucl Med Mol Imaging 2007 34 1427 1438 10.1007/s00259-006-0363-4 17431616

[B9] Schaefer A Kremp S Hellwig D Rube C Kirsch CM Nestle U A contrast-oriented algorithm for FDG-PET-based delineation of tumour volumes for the radiotherapy of lung cancer: derivation from phantom measurements and validation in patient data Eur J Nucl Med Mol Imaging 2008 35 1989 1999 10.1007/s00259-008-0875-1 18661128

[B10] Cheebsumon P Yaqub M van Velden FH Hoekstra OS Lammertsma AA Boellaard R Impact of [(18)F]FDG PET imaging parameters on automatic tumour delineation: need for improved tumour delineation methodology Eur J Nucl Med Mol Imaging 2011 38 2136 2144 10.1007/s00259-011-1899-5 21858528 PMC3228515

[B11] Cheebsumon P van Velden FH Yaqub M Frings V de Langen AJ Hoekstra OS Lammertsma AA Boellaard R Effects of image characteristics on performance of tumor delineation methods: a test-retest assessment J Nucl Med 2011 52 1550 1558 10.2967/jnumed.111.088914 21849398

[B12] Cheebsumon P van Velden FH Yaqub M Hoekstra CJ Velasquez LM Hayes W Hoekstra OS Lammertsma AA Boellaard R Measurement of metabolic tumor volume: static versus dynamic FDG scans EJNMMI Res 2011 1 35 10.1186/2191-219X-1-35 22214394 PMC3285530

[B13] Hatt M le Cheze RC Descourt P Dekker A De RD Oellers M Lambin P Pradier O Visvikis D Accurate automatic delineation of heterogeneous functional volumes in positron emission tomography for oncology applications Int J Radiat Oncol Biol Phys 2010 77 301 308 10.1016/j.ijrobp.2009.08.018 20116934

[B14] Wanet M Lee JA Weynand B De BM Poncelet A Lacroix V Coche E Gregoire V Geets X Gradient-based delineation of the primary GTV on FDG-PET in non-small cell lung cancer: a comparison with threshold-based approaches, CT and surgical specimens Radiother Oncol 2011 98 117 125 10.1016/j.radonc.2010.10.006 21074882

[B15] Yu J Li X Xing L Mu D Fu Z Sun X Sun X Yang G Zhang B Sun X Comparison of tumor volumes as determined by pathologic examination and FDG-PET/CT images of non-small-cell lung cancer: a pilot study Int J Radiat Oncol Biol Phys 2009 75 1468 1474 10.1016/j.ijrobp.2009.01.019 19464822

[B16] Wu K Ung YC Hornby J Freeman M Hwang D Tsao MS Dahele M Darling G Maziak DE Tirona R PET CT thresholds for radiotherapy target definition in non-small-cell lung cancer: how close are we to the pathologic findings? Int J Radiat Oncol Biol Phys 2010 77 699 706 10.1016/j.ijrobp.2009.05.028 19836163

[B17] Hatt M Cheze-Le RC van Baardwijk A Lambin P Pradier O Visvikis D Impact of tumor size and tracer uptake heterogeneity in (18)F-FDG PET and CT non-small cell lung cancer tumor delineation J Nucl Med 2011 52 1690 1697 10.2967/jnumed.111.092767 21990577 PMC3482198

[B18] Boellaard R Krak NC Hoekstra OS Lammertsma AA Effects of noise, image resolution, and ROI definition on the accuracy of standard uptake values: a simulation study J Nucl Med 2004 45 1519 1527 15347719

[B19] Paulino AC Koshy M Howell R Schuster D Davis LW Comparison of CT- and FDG-PET-defined gross tumor volume in intensity-modulated radiotherapy for head-and-neck cancer Int J Radiat Oncol Biol Phys 2005 61 1385 1392 10.1016/j.ijrobp.2004.08.037 15817341

[B20] Steenbakkers RJ Duppen JC Fitton I Deurloo KE Zijp L Uitterhoeve AL Rodrigus PT Kramer GW Bussink J De Jaeger K Observer variation in target volume delineation of lung cancer related to radiation oncologist-computer interaction: a 'Big Brother' evaluation Radiother Oncol 2005 77 182 190 10.1016/j.radonc.2005.09.017 16256231

[B21] Siedschlag C van Loon J van Baardwijk A Rossi MM van Pel R Blaauwgeers JL van Suylen RJ Boersma L Stroom J Gilhuijs KG Analysis of the relative deformation of lung lobes before and after surgery in patients with NSCLC Phys Med Biol 2009 54 5483 5492 10.1088/0031-9155/54/18/009 19706965

